# ACHIEVING THE TRIPLE AIM THROUGH A FALLS PREVENTION INNOVATION IN LONG-TERM CARE

**DOI:** 10.1093/geroni/igz038.1769

**Published:** 2019-11-08

**Authors:** Deanna L Gray-Miceli, Sarah J Ratcliffe, Matthew L Smith, Jeannette Rogowski

**Affiliations:** 1 Florida Atlantic University, Boca Raton, Florida, United States; 2 University of Virginia, Charlottesville, Virginia, United States; 3 Center for Population Health and Aging, Texas A&M University, College Station, Texas, United States; 4 The Pennsylvania State University, University Park, Pennsylvania, United States

## Abstract

Falls disproportionately burden frail older adults in long-term care, which is evidenced by the 60% fall rate annually in this setting. Our health systems improvement model, framed by the Health Outcomes Model for falls prevention, is targeted to achieve the triple aim of better health, better care, and better value. Highlighting evidence from three inter-related studies, this presentation describes the impact of a practice change model and its impact on patient, unit/staff, and organizational level factors as related to facility-wide falls prevention. In study one, we used an evidenced-based practice approach and assessment tool to achieve total and recurrent falls reduction over one year by 32% and 25%, respectively (p<0.001). In study two, the annual savings of using this approach was assessed and showed a total savings of $53,531.00, which equates to $700.00 per falling person. In pilot study three, a convenience sample of 15 older adults who were interviewed about their fall by nurses using this approach stated they felt valued and that the nurse cared about their story (n= 6; 40%). Purposive efforts to embed the model and effective strategies for adoption and implementation are presented.

